# Supplementary material for: Are carbon emissions trading and green financial instruments synergistic? -Comprehensive quantitative research based on content analysis
Source: PLoS One. 2024 Mar 7;19(3):e0298601. doi: 10.1371/journal.pone.0298601 (PMC10919601; doi:10.1371/journal.pone.0298601)
Supplement: S1 Data — (ZIP) [file pone.0298601.s001.zip › Data/地级市Z_F工作报告.zip!/╣π╢1⁄2/├»├√╩╨/└┤╫╘╣1⁄2╓┌║┼í╛┬φ┐╦╩2╛▌═°í┐_2017.docx]

各位代表：

　　我代表市人民政府向大会作工作报告，请予审议，并请政协各位委员和其他列席人员提出意见。

　　一、本届政府工作回顾

　　本届政府任期的五年，是我市历经风雨、爬坡越坎的五年，也是我市砥砺前行、奋发图强的五年。在省委、省政府和市委的坚强领导下，在市人大及其常委会和市政协监督支持下，市政府与全市人民一道，全面贯彻党的十八大和十八届三中、四中、五中、六中全会精神，深入学习贯彻习近平总书记系列重要讲话精神，牢牢把握振兴发展核心要务，认真落实省振兴粤东西北决策部署，积极应对前进道路上的各种困难和风险考验，只争朝夕、主动作为，着力推动全市经济社会持续稳定健康发展，胜利完成十一届人民代表大会确定的发展任务。

　　五年来，我们注重抓项目、稳增长，推动经济发展保持稳中有进、稳中提质良好态势

　　综合实力不断壮大。预计（下同）2016年全市实现生产总值2600亿元，比2011年增长59.9%，总量保持在粤东西北首位，人均GDP突破4万元。公共财政预算收入121.4亿元，比2011年增长83.6%。规模以上工业增加值725亿元，比2011年增长93.9%。在省对粤东西北地区振兴发展评估考核中，2013-2015连续三年居前三位，其中两年第一。粤东西北2015年度县市区振兴指数蓝皮书公布排名，电白区振兴指数位居29个区第一，信宜、高州、化州三市振兴指数居粤东西北45个县（市）前五位。

　　投资规模较快增长。坚持以项目扩投资、增后劲、促发展。五年累计实施市级以上重点项目617个，完成投资998.5亿元，茂名石化炼油改扩建工程、茂名热电厂“上大压小”扩建工程、德国巴斯夫异壬醇等重大产业项目建成投产。2016年全市完成固定资产投资1261亿元，五年年均增长42.1%，总量从全省倒数跃升至粤东西北前列；完成工业投资727亿元，是2011年的8.5倍。

　　产业结构优化升级。三次产业比重由2011年的18.6：39.5：41.9调整为16.7：40.1：43.2。国家级农业标准化示范区数量居全省第一，农业新型经营主体发展迅速。粤西农批建成运营，全球首个桂圆电子交易中心落户高州。建成143万亩高标准农田。2016年茂名石化加工原油1810.5万吨，生产乙烯113.0万吨，茂名在华南地区的石化龙头地位进一步巩固。全市高新技术企业预计增至70家，约为2011年的4.1倍。省级工业园实现县域全覆盖。金融服务发展能力增强，人民币存款余额突破2千亿、贷款余额突破千亿元大关，存贷比五年提升8.2个百分点。2016年旅游业总收入242.4亿元，比2011年增长187.1%。电子商务迅猛发展。

　　五年来，我们注重补短板、打基础，致力改善茂名加快发展的硬环境

　　交通建设全面突破。五年交通建设投资331.9亿元，是我市多年来交通投入最多、发展最快、项目最集中的时期。包茂高速茂名段建成通车，汕湛高速茂名段进入路面施工阶段，云茂高速先行段开工建设，沈海高速鳌头出口开通，高速公路实现从单一线型向网格化发展。深茂铁路茂名段进入铺轨阶段。博贺疏港铁路进入勘察设计招标阶段。市区三座火车站成功更名，站名地理指向得到优化。支持湛江粤西机场迁建工程推进前期工作。先后建成茂名港大道、茂东快线、茂化快线、包茂大道、市民大道二期等项目。改造完成G207、G325及S280、S370等15条国省道。西部快线、潘州大道、工业大道南、市民大道三期开工建设。开通茂南至电白、高州、化州、滨海新区公交。与玉林、梧州两市形成3小时经济圈。形成以中心城区为核心的市域半小时生活圈。

　　环境治理设施逐步完善。建成河西、茂南和高州金山开发区3家污水处理厂，全市污水处理能力合计33万吨/日。茂名石化启动泄漏检测修复技术应用，茂名臻能热电有限公司完成脱硫脱硝治理工程。市生活垃圾焚烧发电厂、各区（市）生活垃圾填埋场建成运营，实现“一县一场”建设目标。109个镇（街）全部建成生活垃圾转运（压缩）站。“城乡清洁工程”五年投入超10亿元。

　　水、电、信息设施加快建设。列入全国规划实施的36宗中小河流治理项目基本完工。高州水库恢复89米淹没处理工程、滨海新区供水工程、水东湾城区引罗供水工程开工建设。电网建设实现质的飞跃，主网220千伏双环网供电格局全面形成，所有镇级配网实现环网供电。“宽带茂名”加快建设。

　　五年来，我们注重拓框架、提品质，大力塑造城市发展新形象

　　中心城区发展空间极大拓展。建成官山五路、西粤北路、文明北路、油城十路、西城西路和官渡三至六路，开工建设西粤南路、站前大道等，北组团东北五小区、油城十路片区、站南片区和西城片区步入全面开发阶段。原茂港区、电白县合并设立电白区。中组团市民高新片区商贸物流项目集聚发展、初具规模。南组团迎宾北路改扩建、水东大道镇东段、环市路和智城大道等加快建设，水东、陈村、高地、南海等片区建设步伐加快。

　　城市人居环境明显改善。北组团完成20多条主干道路及街坊路“黑底化”工程，内街内巷和人行道升级改造加快推进。小东江休闲景观带、官渡公园、红旗公园加快建设。电白城区新建市政道路8条，改造旧城区道路4条，东西湖改造、森高海堤合拢、海堤绿道系统改造和红树林科普教育栈道等项目加快推进。美化绿化亮化水平明显提升。重拳整治城市脏乱差，推进环卫服务市场化改革，城市卫生环境明显改观。完成24宗水浸黑点整治任务。

　　县域城区加快升级开发。信宜市站前片区和城西片区开发成型，打造锦江河“一河两岸”景观带。高州市五年投资超15亿元，新建改建一批重点市政工程。化州市“三大出口”全部打通，“三江六岸”建设水平逐年提升。

　　五年来，我们注重建平台、添动力，全力开创茂名滨海发展新格局

　　滨海新区起步区拓荒前行。新区在一片荒滩上起步，深水大港梦正渐行渐近。东西防波堤项目主体堤身基本完工。通用码头项目动工建设。粤电煤炭码头基本建成。博贺湾大道一期工程建成通车，新城东路、吉达大道、博贺湾大桥及东连接线开工建设。冼太故里景区初具规模，浪漫海岸二期项目建成。

　　高新区迅速发展壮大。积极创建国家高新区。五年建成项目50个，投资64.2亿元；在建项目62个，投资98.2亿元，开发建设面积拓展至12.6平方公里。建成国家危险化学品质量监督检验中心（广东）。高新技术创业服务中心获批国家级科技企业孵化器培育单位。与德国巴伐利亚化学集群签约共建中德（茂名）精细化工园，省编办批复同意高新区管委会加挂该园区管委会牌子。

　　水东湾新城建设稳步推进。三年累计完成土地征收7742亩，实现融资53.8亿元，18项公用设施项目加快建设。水东湾大桥全面开工，海洋公园、歌美海公园、绿道慢行系统有序推进。市中心医院加快建设。高地智慧城路网建设全面铺开。南海旅游岛申报国家4A景区，旅游环境不断改善。

　　五年来，我们注重优环境、保生态，努力践行绿色发展理念

　　环境综合整治扎实有力。主要污染物总量考核保持优秀水平。打赢水东湾渔排网箱清理整治、茂名石化炼油厂卫生防护距离内第一期第一批搬迁安置和乙烯厂卫生防护距离内居民搬迁安置等环保攻坚战。铁腕整治鉴江、小东江和高州水库等江河流域及水库水源保护区污染行为，综合污染指数持续下降，城市集中式饮用水源水质达标率保持100%。黄标车淘汰工作取得阶段性成果。关停河西、公馆片区小炼油、小化工企业116家。市区空气质量指数排名居全省前列。信宜市获批国家生态保护与建设示范区和国家重点生态功能区。茂名滨海公园（第一滩）、放鸡岛分获省“十大美丽海岸”和“十大美丽海岛”称号。

　　绿化茂名大行动持续推进。推进森林围城、进城工程。五年完成碳汇造林44.6万亩，新建高速公路景观林带82公里，国省道绿化1089公里，打造乡村绿化美化示范村809个。自然保护区数量居全省第三，云开山自然保护区晋升为国家级自然保护区。

　　露天矿生态公园建设进展顺利。按照“引水、种树、建馆、修路”思路，连续三年争取国家、省有关资金建设富有茂名特色生态公园，致力留住茂名人的城市记忆。引水工程建成通水，矿区生态修复绿化6208亩，博物馆主体结构完成，环湖、环公园道路全面建成。露天矿区重新焕发生机与活力，生态环境明显改观，极大地促进了北组团空气质量的改善。

　　五年来，我们注重抓改革、破难题，有力振奋了全市上下抓发展的精气神

　　供给侧结构性改革等深入推进。出台并推进供给侧结构性改革总体方案和五个行动计划。统筹推进放管服改革。市县镇村四级联动网上办事大厅建成投用，我市被列为2016年全国政务服务标准化试点市。深化商事登记制度改革，全市市场主体比2011年增长90.8%。大额资金使用、非税收入管理、政府性债务管理纳入制度化轨道。“营改增”改革和机关单位公车制度改革全面完成。完成茂名港集团、交投集团、水投集团、城投集团等国企组建。

　　要素瓶颈制约有效突破。推广征地拆迁“大包干”和“六个一”工作机制，滨海新区起步区、高新区推行区镇互动体制改革，重点项目用地得到保障。突破一批长期制约项目建设的用林、用海难题。抓住土地利用总体规划中期调整机遇，新增用地规模居全省中上水平。开拓直接融资渠道，成功发行企业债券和中期票据，PPP融资模式逐步推广。两年招聘急需紧缺人才57名。

　　开放发展迈出新步伐。对内对外合作取得新突破。抢抓包茂高速开通机遇，与广西梧州市签订战略合作框架协议。积极落实“一带一路”国家战略，组团出访德国、荷兰开展经贸文化交流活动。经济外向度不断提高，外贸进出口总额突破100亿元。

　　五年来，我们注重办实事、惠民生，有效增强人民群众获得感和认同感

　　社会保障体系日趋完善。五年民生支出1095.8亿元，占财政总支出的80%，年均增长18.6%。就业创业形势较好，五年新增就业35.4万人，新增转移就业劳动力54.5万人。建成3个创业孵化基地。五年扶持妇女创业小额担保财政贴息贷款5137人、2.9亿元；2015年以来发放创业担保贷款587笔、8680万元。城镇职工人均养老金提高至2061元/月。实施城乡居民大病保险制度。城乡居民基本医保支付比例平均提高10个百分点，年度最高支付限额（含大病保险）提高至38万元。养老服务事业走在粤东西北前列。城乡低保、五保救助和孤儿、残疾人基本生活保障水平大幅提高。五年开工建设公共租赁住房5938套、棚户区改造8134套，完成农村危房改造4.3万户。

　　社会事业加快发展。坚持创新驱动发展，五年获省科技奖27项，居粤东西北前列。建成3个国家级众创空间。被列为国家知识产权试点城市，发明专利申请年均增长率居全省前列，全市研发经费投入年均增长27.9 %。成功创建广东教育强市，各区（市）高标准通过国家义务教育发展基本均衡县（区）认定。建成茂名一中新校区和愉园中学、愉园小学，开工建设育才学校。“一本五专”高等教育格局加快形成，广东石油化工学院西城校区开工建设，广东茂名健康职业学院、幼儿师范专科学校成功开办，茂名职业技术学院新校区、广州科技职业学院滨海校区加快建设，广东茂名农林科技职业学院获批筹建。建成市青少年活动中心。精神文明建设深入推进，大力弘扬社会主义核心价值观和新时期茂名精神。文化事业繁荣发展。成功创建“中国硬笔书法名城”。群众性文艺创作走在全省前列，基层公共文化设施实现全覆盖。群众体育活动蓬勃开展。医药卫生体制改革继续深化，公共卫生服务能力持续增强，中医药强市工作扎实推进。

　　社会治理深入推进。聚焦创建“平安茂名”，织牢全市立体化治安防控体系，重点地区、重点部位和重点时段治安防控全面加强，“全民创安”社会治安防控体系建设经验在全省推广。严厉打击电信诈骗、飙车、涉毒、涉枪等突出违法犯罪行为，治安重点地区整治获公安部充分肯定，社会治安形势持续向好。网络空间日趋清朗。突出抓好危化品生产存储经营、道路交通、油气管道、公安消防领域安全管理，安全生产形势保持稳定。突发事件应急联动能力进一步提升。食品药品安全管理不断加强。依法治市步伐加快，率先在全省开展“一村（居）一律师”工作。同时，人口计生、妇女儿童、民族宗教、国防动员、残疾人、统计、双拥、气象、地震、外事、侨务、三防、人防、打假等工作都取得新成绩。

　　我们高度重视政府自身建设。把遵守宪法和法律作为施政根本原则，建立政府常务会学法制度，依法决策、民主决策水平不断提高。主动接受人大政协监督和社会舆论监督。政府立法工作开局良好，第一部地方性法规《高州水库水质保护条例》由市人大常委会审议通过并获省人大常委会批准实施，出台第一部政府规章。建立市县镇三级政府权责清单制度。依法办理行政复议案件500多件。切实加强政务公开，推进网络问政。不断强化监察、审计监督作用。驰而不息落实中央八项规定，坚决纠正“四风”，抓好“三严三实”专题教育、“两学一做”学习教育，筑牢纪律防线，反腐败斗争形势持续向好。

　　各位代表！经过五年的不懈努力，我们不仅有效应对了各种风险挑战，还有力提升和发展了茂名，也塑造了茂名迎难而上、奋力拼搏的良好形象。我们深深体会到，千方百计、持之以恒抓落实，是我们取得所有成绩的第一法宝，必须长期坚持、不断发扬：一是创新机制抓落实。大胆创新落实工作机制，整合资源、协调各方、联动配合，形成责任明确、措施明确、时间明确的倒逼落实新机制。二是考核激励抓落实。建立完善“两会两考两通报”制度，给干部赛场，用结果说话，激发工作动力，形成你追我赶良好氛围。三是强化督查抓落实。建立大督查工作格局，持续加大督查投入、强化督查力量，把重要决策、重点工作、重大事项全面列入督办范围，全力做到事事有落实、件件有回音，不放空头炮。四是敢于突破难点抓落实。在敢啃“硬骨头”、敢碰硬钉子上下功夫，坚决推进一批悬而未决、久拖不决的重难点事项，以难点的不断突破，推动形成更大发展局面。五是以钉钉子精神抓落实。对重点工作和重要部署紧追不放、反复督办，不见成效不收兵，最大限度确保落实、推进工作、促进发展。

　　各位代表！过去五年极不平凡，成绩来之不易。这是省委、省政府和市委正确领导的结果，是全市人民团结拼搏、共同奋斗的结果。在此，我代表市人民政府向全市人民，向驻茂人民解放军、武警官兵和各民主党派、工商联、人民团体、社会各界人士致以崇高敬意！向长期关心支持我市发展的港澳同胞、台湾同胞、海外侨胞及国际友人表示衷心感谢！

　　我们也清醒地认识到，我市改革发展仍存在不少矛盾和问题：发展的基础和条件依然薄弱；发展框架拉开以后，要素需求增加与供应难度加大的矛盾突出；产业结构过于单一，抗风险能力较低；城镇化水平不高；营商环境有待改善；发展亟需的各类专业化人才不足；民生欠账较多；改进作风、反腐倡廉仍然任重道远。对这些问题，我们将高度重视，采取有效措施认真解决。

　　二、今后五年奋斗目标和主要工作任务

　　今后五年，是茂名全面实现“十三五”发展规划和全面建成小康社会攻坚阶段，对茂名的发展至关重要。市第十一次党代会为茂名的新发展描绘了美好蓝图。我们要认真贯彻党代会精神，全面落实“一体两翼三大抓手”发展思路，全面实施“三个做大”发展战略，牢固树立创新、协调、绿色、开放、共享发展理念，加快推动茂名振兴发展。

　　今后五年的主要目标是：到2021年，生产总值突破3300亿元，年均增长8.5%，人均生产总值年均增长7.5%，城乡居民收入增长与经济增长同步，如期全面建成小康社会。

　　实现上述目标，我们要着力抓好六方面工作：一是打造特色鲜明产业强市。培育壮大主导产业，形成新的产业增长点。重点抓好石油化工、农副产品加工、矿产资源加工、特色轻工纺织、医药与健康、金属加工及先进装备制造六大主导产业。依托“三大平台”打造发展新增长极。二是打造粤西重要交通枢纽。完善“四横两纵”铁路网。建成“三横三纵”高速公路网。打造综合性深水大港。完善提升主干公路网，推进城市组团交通一体化，建设现代客货站场。同时，构建现代能源保障体系，着力建设现代水利保障体系。三是打造宜居宜业滨海绿城。建设粤西组团式中心城市，推进北中南东四大城市组团建设。优化发展现主城区，中联发展市民高新片区，南拓发展水东湾城区，东进建设博贺湾片区。争创全国文明城市。四是壮大特色县域经济。壮大县域产业规模，提升发展质量，推进县域新型工业化进程。加快转变农业发展方式，发展现代农业。加快城镇化步伐，形成布局合理的城镇体系。推进社会主义新农村建设，建设茂名特色美丽乡村。五是深化改革开放。坚持向改革要动力，突出抓好行政管理体制、经济体制、金融改革创新。参与“一带一路”建设，加强区域合作，推进外经贸转型升级和招商引资工作。六是推进民生持续改善。坚守民生底线，大力推进基本公共服务均等化。加快发展科技、教育、就业、社保、医疗、扶贫开发等各领域事业。加强生态环境保护，加强和创新社会管理，促进社会和谐稳定。

　　三、2017年工作安排

　　2017年是新一届政府开局之年，也是“十三五”规划全面实施的关键一年，做好今年政府工作意义重大。综观国内外形势，我们面临的发展环境更加错综复杂，挑战与机遇并存。一方面，世界经济深度调整，国内经济仍然面临下行压力，区域竞争日趋激烈并呈现分化，我市缺乏大的新的经济增长点；另一方面，国际原油价格预期温和回升，积极财政政策仍将持续，发达地区产业转移加快，新产业新业态蓬勃发展，我市交通等基础设施短板逐步补齐，发展框架全面拉开。总体而言，我市发展仍处于重要战略机遇期，我们要认清形势、坚定信心，锐意改革创新，奋力攻坚克难，确保新一届政府开好局、起好步。

　　今年政府工作总体要求是：全面贯彻党的十八大和十八届三中、四中、五中、六中全会和中央经济工作会议精神，深入学习习近平总书记系列重要讲话精神，认真贯彻落实省委十一届七次、八次全会和市十一次党代会精神，强化五大发展理念，坚持“稳中求进”总基调，以供给侧结构性改革为引领，紧紧扭住“三大抓手”，培育壮大主导产业，加强交通设施建设，推动城市扩容提质，狠抓改革创新，提升开放水平，促进绿色发展，加强民生保障，为加快茂名振兴发展奠定更加坚实的基础。

　　今年经济社会发展主要预期目标是：生产总值增长7.5%左右，固定资产投资增长15%，社会消费品零售总额增长10%，一般公共预算收入增长6%，居民消费价格涨幅控制在3%以内；居民收入增长速度高于经济增长速度。

　　今年重点抓好以下八方面工作：

　　（一）突出培育壮大主导产业，不断增强经济实力和竞争力

　　把发展实体经济作为今后一段时期工作的重中之重，努力打造特色鲜明产业强市。

　　培育壮大工业主导产业。实施《培育壮大工业主导产业五年行动计划》，推动形成六大特色优势主导产业集群。积极加强与茂名石化沟通合作，争取更多项目和原料布局茂名，延伸中下游产业链，推动石化产业做大做强。推动博贺新港区至高新区、茂南石化工业园区化工管廊项目建设。争取成品油战略储备项目和甲醇制烯烃项目落地。加快推进LNG项目前期工作，动工建设LNG造陆项目。加快实施重点骨干企业培育计划。建立主导产业重点建设项目库，实行分级动态监测管理。支持围绕主导产业设立和完善行业协会。强化产业发展在各区（市）经济发展考核中的权重。加大对主导产业项目建设、投融资服务、用地（林、海）、品牌奖励等支持力度。积极创建质量强市示范城市。

　　抓好园区提质增效。推动产业向园区集中。积极探索园区市场化、企业化管理新模式，大力推进通用厂房建设。推广产业用地“先租后让”供地方式，完善租赁土地厂房项目报建手续。完善园区管理机构和投资开发公司双重架构。进一步创新省级园区管理体制，实现园区事务在园区办结。加强人才交流和引进，提升园区用地、融资和经营水平。举全市之力支持茂名高新区创建国家高新区。全力推进中德（茂名）精细化工园建设。加快博贺新港区物流园、临港工业园和健康产业园的规划建设。整合高岭土资源开发，建设高岭土产业园。统筹发展好茂南石化工业园。

　　大力发展现代服务业。推动市民高新片区商贸物流重点项目建设。加快规划建设茂名粤西空港经济区。促进农村商品流通网络建设。大力支持电商经营主体和电商产业园（物流园）发展。大力发展文化体育产业。推动健康和养老服务业发展。落实《茂名市鼓励金融业支持地方经济发展实施办法》。加快农信社向农村商业银行转制改革。继续引进股份制商业银行，鼓励发起设立村镇银行。创新政银企合作模式，引导金融机构创新产品和服务。完善政府主导融资性担保机构，扶持民营融资担保机构。壮大中小微企业信贷风险补偿金规模。深入推进农村和中小微企业信用体系建设。支持中小微企业直接融资。推动政策性保险业务开展。

　　着力打造特色旅游品牌。结合茂名实际完善旅游发展规划。突出“泉、海、岛”滨海旅游特色。支持南海旅游岛申报国家4A景区。努力建设南中国避寒、避暑养老基地。全面推进乡村旅游，再推出10个“美丽乡村”旅游项目。支持电白区、信宜市做好创建省级全域旅游示范区工作，完善旅游基础设施及配套项目。打造冼太文化旅游品牌。创新旅游宣传推介方式，加强与旅游服务电商平台合作，创建“互联网+旅游”新模式。

　　（二）突出交通基础设施建设，持续改善发展硬环境

　　抓好面向未来的基础设施建设，构建粤西重要交通枢纽，补齐能源、信息、水利等基础设施短板，提高经济社会发展承载力。

　　畅通交通“大动脉”。建成汕湛高速茂名段。全面开工建设云茂高速茂名段。力争启动沈海高速扩建工程。基本建成深茂铁路茂名段。开工建设博贺疏港铁路项目。推进河茂铁路、茂湛铁路电气化改造工程建设。

　　疏通交通“毛细管”。完成S291线公馆油库至七迳段改造工程。建成西部快线、工业大道南段、潘州大道。开工建设南排大道、中德大道、茂化快线延长线（高山至公馆旧村段）。加快完善通达县城、中心镇及重要旅游景区的高速公路连接线。推进市域县乡公路和农村公路建设。

　　升级交通“关节点”。加快茂名火车站南侧站房、广场等配套建设。积极推进茂名东站现代铁路货场项目，扩建茂名西站铁路货场。完成茂名港博贺新港区防波堤配套工程建设，加快东区化工仓储、广港通用码头建设，积极推进30万吨级航道及原油码头、10万吨级成品油码头、油气化工码头、利丰散货码头、港政码头、昌利液体化工码头及配套仓储项目等项目前期工作。

　　力补基础设施短板。继续实施中小河流治理工程。加快滨海新区供水、水东湾引罗供水、高州水库除险加固工程89米正常蓄水淹没处理和海堤达标加固等工程建设。推进自来水应急管网、河东水厂扩容、城区旧管网改造、第一污水处理厂提标扩容改造及河东片区污水总管等项目建设。加快电网和信息基础设施建设。

　　强化交通、水务管理。打击超限超载违法运输行为。加强公路沿线管理，遏制违法建筑、非法广告标志设施设置行为。取消公路车辆通行费年（次）票制。全面建立“河长制”，加强河道、采砂和水资源、水环境监管。

　　（三）突出中心城区扩容提质，切实加快城市化进程

　　坚定不移拉大城市框架，推动中心城区向东向南靠海发展，促进组团间融合发展，致力打造宜居宜业滨海绿城。

　　拓展优化北组团。加快推动东北五小区、油十片区、站南片区及西城片区开发建设。抓好重点村庄“三旧”改造，有序推进棚户区改造。加快推进茂名石化炼油厂卫生防护距离内居民搬迁安置。加快人民北路、大园路、站前大道余段、金城路、站南路、红旗北路延长线项目建设。推进高凉南路、高凉北路、西粤北路、江东北路等新建道路项目。完成茂苍路改造以及迎宾三四路、计星南路沥青罩面改造工程。推进人行道和小区道路升级改造。加强黑臭水体整治。做好茂南大道污水总管建设和站北六七路、西粤南路污水管改造工作。加快推进官渡、站南、钟鼓公园建设。推动特色街区建设。完善小东江综合整治相关工程。大力提升中心城区亮化绿化水平。完成国家园林城市复查工作。

　　着力发展中组团。抓好国家新型城镇化试点工作。推进商贸物流项目集聚发展，发展都市产业园。加快推进高新客运站建设。加快建设石化产品运输车辆综合服务中心。推进综合污水处理项目建设。推进德国风情小镇、雨公山森林公园建设。

　　加快建设南组团。加快实施电白城区“八路一堤一广场”市政建设。抓好水霞路、兴华路、登步路、环湾路等道路建设。加快水东湾综合整治开发。继续大力推进水东湾大桥、歌美海大桥及新城路网建设。加快南海旅游岛配套设施建设。推进高地智慧城学校、医院、公园等项目建设。

　　统筹建设东组团。完成茂名港大道改线段、博贺湾大道、原G325电城段路面改造、污水处理厂（首期）工程。基本完成博贺湾新城东片区土地一级开发、公租房配套设施工程。加快博贺湾大桥及东连接线、茂名港大道延长线、新城东路、吉达大道建设。开工建设高州人民医院滨海分院、综合管廊试点和污水管网工程。

　　提高城市精细化管理水平。启动创建国家卫生城市和全国文明城市工作。加强城市管理委员会统筹协调作用。推动城市管理从主干道向大街小巷延伸。加强城市交通秩序综合治理。抓好治摩治超及占道经营、“牛皮癣”治理等工作。落实好“门前三包”责任制。加强城市社区治理和小区物业管理。加快农贸市场、停车场改造和布局，规范户外广告经营管理。严厉打击违建行为。实施数字城管工程，推动智慧城市建设。

　　（四）坚持开放发展，着力集聚振兴茂名资源要素

　　在改善交通条件和拓展发展平台基础上，依托特色资源和优质服务，全方位扩大开放，汇聚加快茂名振兴发展的资源要素。

　　加大招商引资力度。各级主要领导带头招商。落实好促进民营经济发展政策措施，大力支持民营经济发展。建立健全招商引资工作联席会议制度。加强惠企政策宣传和落实。完善招商项目库，实施靶向精准招商。推进本地和异地商会建设，借力商会招商。主动对接珠三角龙头企业和上下游配套企业，实施产业链招商。积极主动作为，用好用足省产业共建政策，推动与珠三角一体化发展。加大劳动密集型企业招商引资力度。

　　大力优化营商环境。畅通企业诉求渠道，落实联系企业制度。落实并联审批。简化技改项目、中小项目和改扩建项目审批流程。注重培养行业审批专业人才，切实增强紧缺岗位审批力量。完善招投标流程，严把资格预审和评标关。严格落实国家和省全面清理规范涉企收费措施，清理规范涉企经营服务性收费、行政审批中介服务收费。继续推进售电侧改革。落实小微企业税收优惠政策和国家高新技术企业减免税政策。完善物流配送体系，推进物流标准化和通关便利化，进一步降低企业物流成本。

　　加强对内对外交流合作。抓住交通条件改善机遇，加强区域合作，向东强化与珠三角经济联系，向北积极开拓包茂高速公路沿线城市经贸市场，深化茂名梧州战略合作及环北部湾城市群合作，加快打造茂湛阳沿海经济带，推进茂名玉林战略合作。抢抓“一带一路”机遇，深化与德国、荷兰等国友好城市交流合作，拓展与日本及东南亚国家交流合作。加快口岸建设。支持跨境电子商务发展。多渠道多形式组织外贸企业参加重要经贸活动。支持六韬珠宝创意产业园发展，促进外经贸工作上新台阶。

　　（五）坚持统筹协调，着力构建城乡发展新格局

　　在强化滨海发展战略的同时，坚持统筹兼顾，山海联动、城乡互动，推动茂名区域和城乡协调发展。

　　突出抓好“三大平台”建设。滨海新区起步区继续强力推进博贺新港区建设，加快启动临港工业区建设，注重抢抓新兴产业发展机遇。高新区着力推动从传统工业园区向现代工业园区转变，从单纯工业园区向产城融合片区转变。水东湾新城注重依托电白城区扩张和自身产业成长，加快高地智慧城和南海旅游岛建设。

　　统筹县域经济发展。各区（市）要树立标杆，对照标杆学经验找差距。因地制宜发展特色主导产业。积极引入民间资本，推动工业园区、电商产业园、企业孵化园等县域产业平台建设。着力补齐交通、水利、环保、信息等基础性短板，强化县域发展基础。加强教育、医疗、文化体育等公共设施建设，完善各类生活配套设施，增强县城承载力和集聚力。修编各县域副中心及重点中心镇城镇总体规划，加快建设县域副中心。提升各县城至县域副中心和中心镇公共交通服务水平。提高城镇综合管理水平。加快推进一批特色小镇、专业村镇规划建设。

　　推进农业供给侧结构性改革。扶持发展农业龙头企业、农民专业合作社、家庭农场、专业大户等新型经营主体。大力提升种植规模化水平，加快特色农产品产业化进程。扶持壮大罗非鱼产业。加强深水网箱养殖发展，做好水产健康养殖示范工作。做好渔船减船转产和更新改造工作。大力发展休闲观光农业、生态农业。

　　加快新农村建设。强化农村规划管理。加快建设高标准基本农田和耕地提质改造工作。推进农村集体土地承包经营权确权登记工作。完善农村公共服务平台、集体“三资”管理系统建设。加快推进茂南、信宜、化州3个省级新农村示范片建设。高标准建设10条美丽水库移民村。加快实施村村通自来水工程。实现农村电子商务服务站在行政村全覆盖。

　　（六）坚持改革创新，着力增强发展新动力

　　大力实施创新驱动发展战略，解放思想、深化改革，优化创新环境，打造创新平台，激发创新活力。

　　不断深化行政体制改革。深化简政放权、放管结合、优化服务改革。推进市场准入负面清单制度改革、行业协会商会与行政机关脱钩专项改革。切实抓好“互联网+政务服务”改革，加快政务服务标准化建设，推进市级“一门式”公共服务暨公共资源交易服务实体大厅合作共建。规范和促进政务信息资源共享，推进大数据平台建设。全面实施不动产统一登记制度。开展企事业单位公务用车制度改革。突出抓好城市综合执法体制改革。深入推进事业单位改革和商事制度改革，推进审计、环保管理体制改革。

　　深入推进供给侧结构性改革和国资监管、财税体制改革。切实抓好“三去一降一补”。稳步推进去产能工作，切实做好地方政府债务置换，积极推进房地产去库存。加快完善国有资产全覆盖监管格局。推进国有企业重组，探索组建金控集团和幼教集团。在基础设施和公用事业领域加大政府和社会资本合作（PPP）模式推广力度。加快推进财政体制调整和预算管理改革，加强重大项目资金管理和政府性债务限额管理。深化国库管理和国地税征管体制改革，全面推进国地税共建服务大厅工作。

　　加快创新主体培育。促进科技与金融紧密结合，强化知识产权服务，加快培育发展高新技术企业。加强产学研合作，推进我市与14家签约高校、科研院所全面合作，支持广东石油化工学院建设高水平应用型大学。培育壮大众创、众包、众扶、众筹等创新创业新模式。实施农民工、大学生和退役士兵等人员返乡创业支持计划。继续推进新一轮工业企业技术改造。组建一批新型研发机构、工程中心、重点实验室等创新平台，支持茂名市气象台申报国家级创新平台。加快科技企业孵化器和众创空间建设。

　　强化创新人才支撑。完善高层次人才引进培养和奖补政策规定。通过购买服务、加强项目合作等多种形式，引进优秀创新创业人才团队。积极实施省“扬帆计划”和“特支计划”，吸引高层次人才携技术项目来茂名创新创业。加快建设高层次人才公寓。注重人才结构平衡，统筹引进高端人才、中初级人才、工匠技能人才和社会人才。加强青年科研人才和科研后备力量建设。实施企业领军人才培养工作。

　　（七）坚持绿色发展，着力打造山清水秀生态环境

　　牢固树立“绿水青山就是金山银山”的发展理念，把生态环保工作作为重大民心工程抓紧抓好。

　　推进生态建设工程。积极争创国家森林城市。继续推进绿化茂名大行动。完成70个森林家园、18个森林公园、4个湿地公园和200个乡村绿化美化示范点建设。推动信宜国家重点生态功能区建设。探索道路两侧绿化新机制。加快百里银杏长廊和包茂高速公路等道路两侧生态景观林带建设。启动建设彭村湖生态湿地公园。抓好露天矿生态公园品质提升。加强沿海防风林、红树林及河口湿地生态功能区保护。推进美丽海湾海岛建设。完成国有林场改革。

　　抓好节能减排。落实节能减排指标任务。加快淘汰落后产能。支持企业利用新技术、新标准开展清洁生产。加快推进循环经济示范中心项目建设。实施重点节能工程。加快淘汰黄标车和禁燃区内燃煤小锅炉。实行公共交通优先，推广新能源汽车。

　　加强环境综合治理。按照“截污水、关猪场、打偷排”思路，抓好小东江、鉴江、袂花江等重点河流污染治理，提高交界断面水质达标率。抓好高州水库、罗坑水库等重点湖库水源水质和生态保护。加快污水处理设施及配套管网建设与改造。加强农业面源污染防治、畜禽养殖污染整治和农村环境综合整治。抓好重点区域、重点月份和特殊天气空气质量预报预警和应对。推进重点行业挥发性有机物（VOCs）监测能力建设和综合整治。加强环境监管执法。深入开展“城乡清洁工程”。稳步推进县域绿能环保发电项目建设。

　　（八）坚持共享理念，着力提升民生和社会事业发展水平

　　着力补短板、兜底线、抓稳定、保平安，坚持共享普惠，提升民生福祉。

　　筑牢社会保障线。加大社保扩面征缴工作力度。稳步推进社保城乡一体化改革和机关事业单位养老保险制度改革。提高底线民生保障水平，完善多层次养老服务体系。抓好充分就业工作，全年新增城镇就业6万人，促进创业2200人，转移劳动力技能培训1万人。促进职业技术学校、高校人才培养与企业需求有效对接。深入推进精准扶贫，全年实现脱贫6.5万人。实施农村贫困户危房改造2.3万户，开工建设棚户区改造住房702套，基本建成各类保障性住房457套。全力提升城乡防灾减灾能力，抓好县级救灾仓库建设工作。

　　打好民生实事牌。落实好省十件民生实事各项任务。开展广东省推进教育现代化先进市创建工作，加快“一本五专”高等院校建设。建成育才学校，启动建设崇文学堂。鼓励发展民办教育。扎实推进卫生强市建设。全面推进城市公立医院改革，完成乡镇卫生院业务用房改扩建工作。加强职业卫生应急救援能力建设。加快养老福利设施建设。抓好人口计生工作。继续实施农村“三关爱”工程。开展基层文体广场和综合性文化服务中心示范点建设工作。传承弘扬冼夫人文化。加快发展体育事业，推进全民健身活动。深化殡葬改革。

　　构建辖区安全网。切实推进“平安茂名”建设，严防妥处暴恐活动，重拳打击飙车、涉枪、涉毒、电信诈骗等各类突出犯罪。构建立体化社会治安防控体系。着力推进中心城区治安和交通管理。加强农村基层治安治理。落实安全生产党政同责、一岗双责、齐抓共管、失职追责，抓好重点领域事故隐患排查整治，坚决预防和遏制重特大事故发生。加强网络安全监管。推进基层治理创新，抓好“一村（居）一律师”和“以案定补”工作。全面推进食品药品“三级”网格化监管。切实做好统计、工青妇、民族宗教、国防动员、科学普及、外事、侨务、双拥、人防、气象、地震、残疾人、档案、红十字、方志、文史等工作。

　　打铁还须自身硬。做好今年经济社会发展各项工作，必须加强政府自身建设。一是切实加强民主法治建设。自觉接受人大及其常委会监督，认真落实其各项决议、决定并定期报告工作。自觉接受人民政协的民主监督，接受社会和舆论监督。办理好人大代表建议和政协委员提案。发挥好专家学者思想库和智囊团作用，提高决策科学化水平。做好冼夫人历史文化保护、露天矿生态公园保护、化橘红中药文化保护、城镇公共停车场管理和禽畜养殖管理等立法工作。加强法治政府建设，提高依法行政水平。二是着力抓好作风建设。构建“亲”“清”新型政商关系，支持引导商协会蓬勃发展。发扬钉钉子精神，敢于啃“硬骨头”，牢牢盯住重点工作和重点项目，致力把既定目标和蓝图变为现实。强化责任担当和督查问责。加强审计监督。进一步完善考核激励机制。落实“三个区分”，鼓励勇于担当敢于作为。推进网络问政深入开展。三是持续推进廉政建设。贯彻《中国共产党党组工作条例（试行）》，落实《关于新形势下党内政治生活的若干准则》和《中国共产党党内监督条例》，筑牢拒腐防变思想防线和制度防线，致力营造干净干事良好氛围。

　　各位代表！站在新起点，展望新征程，我们信心满怀！让我们紧密团结在以习近平同志为核心的党中央周围，在省委、省政府和市委的正确领导下，锐意创新、攻坚克难、埋头苦干、奋发图强，不断谱写茂名振兴发展新的篇章！
